# Target familiarity and visual working memory do not influence familiarity effect in visual search

**DOI:** 10.1038/s41598-021-86669-2

**Published:** 2021-04-07

**Authors:** Zhihan Guo, Maolong Niu, Qi Wang

**Affiliations:** 1 State Key Laboratory of Nuclear Power Safety Monitoring Technology and Equipment, China Nuclear Power Engineering Co., Ltd., Shenzhen, 518172 China; 2 Department of Psychology, Sun Yat-Sen University, 132 Waihuan Donglu, Higher Education Mega Center, Guangzhou, 510006 China

**Keywords:** Psychology, Human behaviour

## Abstract

Familiarity effect refers to the phenomenon that searching for a novel target among familiar distractors is more efficient than that searching for a familiar target among novel distractors. While the familiarity of distractors is considered as a key role on familiarity effect, the familiarity of targets contribute to this asymmetric visual search is unclear. The present study investigated how target familiarity influences visual search efficiency from the perspective of perceptual load. Experiment 1 using two similar Chinese characters (“甲” and “由”) suggested that searching for a familiar target from familiar distractors is an inefficient search process in Chinese context. Experiment 2 adopted a dual-task paradigm with a visual working memory task to increase the perceptual load and attempt to affect the efficiency of searching a novel target (mirrored “舌”) from familiar distractors (“舌”). Results demonstrated no difference in the search efficiency between single and dual-task conditions. The present study suggests that the familiarity of target does not influence the search efficiency with familiar distractors when involving semantic processing of Chinese characters. Additionally, the interference of extra working memory load would not impair the efficiency of searching target among familiar distractors, supporting the critical effect of distractor familiarity on the efficiency of visual search.

## Introduction

**Figure 1:**
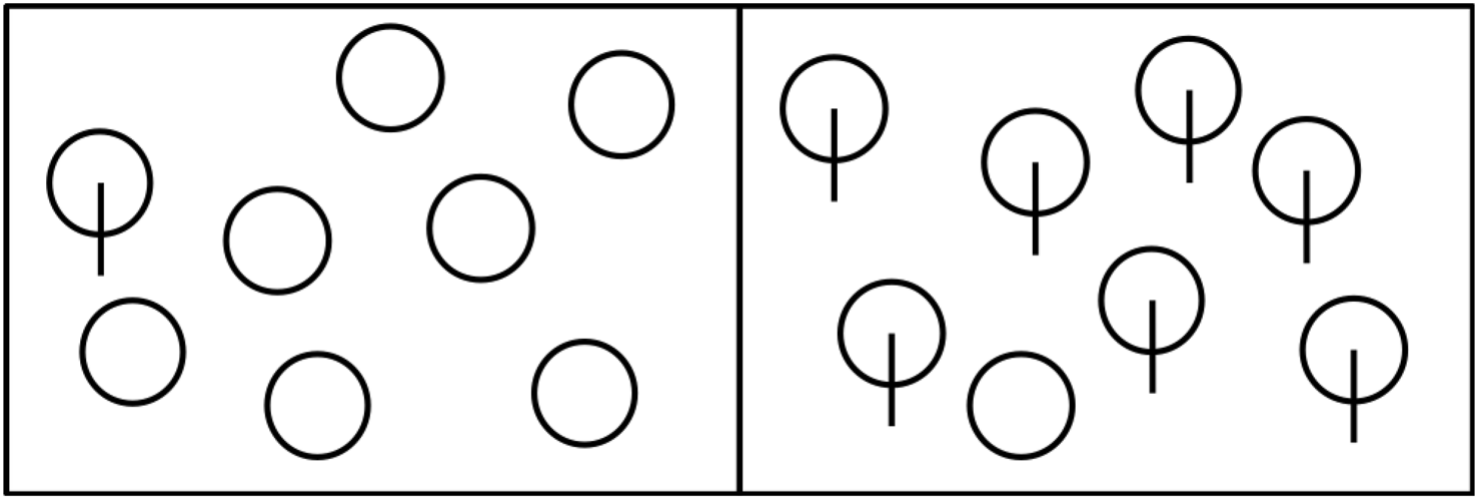
It is easier to find the circle with the vertical line among plain circles (left panel) than vice versa (right panel).

Visual search is a commonly used paradigm to explore attention mechanisms, which also occurs very often in real work or daily life. A typical visual search task requires subjects to seek a target in the stimulus series comprising distractors and targets. The search efficiency is generally represented by the slope of the function of response time regarding the number of stimulus items. Haslam et al.^1^ proposed that a continuum of search efficiency was a better way to understand attentive processing than in terms of a dichotomy corresponding to serial and parallel search. A typical indicator for efficient search is the slope that is less than 10 ms per item^2^. Visual search asymmetry proposed by Treisman and Souther^3^ refers to the phenomenon that, given two visual stimuli A and B, there is a significant difference in search efficiency between searching for B among A and searching for A among B. They developed a classical paradigm for studying search asymmetry. As shown in Fig. 1, searching for a circle with line segments among circles is a very efficient (left panel), while searching for a circle among circles with line segments is less efficient (right panel).

Generally, human memory may have stored mental representation of commonly encountered or learned information. Comparing with unfamiliar items, the mental representation of familiar ones may facilitate the processing from both the perceptual and conceptual perspectives^4^. Searching for familiar objects in daily life has been suggested to be faster than unfamiliar ones in visual search tasks^5^. Meanwhile, we hold a high sensitivity to novel events is a universal phenomenon with evolutionary significance^6,7^. Many studies have found that visual search for a novel target among familiar distractors is more efficient than searching for a familiar target among novel distractors^8–10^. This phenomenon of visual search asymmetry is known as the familiarity effect. The results of Meinecke and Meisel^11^ support robustness of such familiarity effects: even if the density and number of familiar distractors (N) are increased, the novel target (И) still shows pop-out effects. When the target appears at the edge of the field of view that impairs letter recognition, the familiarity effects is still observed, indicating that familiarity effect rely less on bottom-up processing^11^.

To explore the mechanism of familiarity effect, visual search studies have varied the familiarity of targets and distractors respectively to examine the factors that influence the search efficiency. As for the search asymmetry with the advantage of familiar distractors, researchers proposed that the novel target may become more salient when searching from familiar distractors, as familiar distractors might be processed in a combined way^12,13^. Neurological evidence supported the process of inhibiting distractors and highlighting target during visual search. For example, humans and monkeys can locate a target faster among familiar distractors, with the inferotemporal cortex less activated by familiar distractors and activated more by targets^5^. Meanwhile, familiar distractors can be quickly processed, while a novel target may lead greater activation in the brain, thus attracting more attention^14^. Other research suggested that familiarity effect may result from the difference in familiarity between the target and distractors^9^. One event-related potential study showed that the visual mismatch negativity component (vMMN) produced by a mirror N (namely И) as the searching target has a shorter latent period compared with that of N. The results supported that human brain may be more sensitive to the novel target when searching from familiar distractors^15^. Malinowski and Hübner^13^ recruited two groups of participants that had different familiarities with N and И. One group of participants were from Germany, who are familiar with N but unfamiliar with И. The other group consisted of Slavic participants, who are familiar with both N and И. The results showed that for the German group that was only familiar with N, searching for the novel И among Ns was very efficient, while searching for N among the novel Иs was difficult. However, for the Slavic group with familiarity with both N and И, both types of search were parallel and there was no significant difference in search efficiency. They concluded that search efficiency is only related to the familiarity of distractors but has little correlation with the familiarity of the target, which supports the accounts of Karni and Sagi^12^.

However, the theoretical account that only the familiarity of distractor contributes to familiarity effect has been challenged by relevant research using various types of searching materials. Firstly, the familiarity effects with N and И stimuli were more dramatic than other letter stimuli, such as P, K, or F^10^. Studies used uppercase letters with similar visual perceptual features to construct familiar-target and familiar-distractor pairs, like C/G, E/F and P/R, failed to repeat the highly efficient search as with letter N and И^16^. The study also showed that although searching for a familiar target among novel distractors presented steeper slopes than that of searching among familiar distractors in the target-present trials, the search slopes in the target-absent trials did not differ between novel distractor and familiar distractor conditions^16^. This result indicated that search asymmetries cannot be explained solely by the rapid process of rejecting familiar distractors.

Secondly, when searching items involve complex perceptual features and even semantic meanings, the familiarity of target could also influence the search efficiency. For instance, Mruczek and Sheinberg^17^ used images of daily objects as searching materials, such as lamps, cars, chairs, butterflies, teddy bears, and bamboo baskets. They asked participants to familiarize themselves with images for 4 or 5 days 1 week before performing the search task. Even though the study consistently showed that familiar distractors can promote the visual search, the efficiency of searching for a novel target was lower than that of searching for a familiar target. In the same vein, the search of upright Stars and Stripes flags was significantly more efficient than of inverted flags^18^ and also for highly familiar logos^19^. Tong and Nakayama^20^ found a crossover pattern of search asymmetry for face stimuli. Target identification was faster for the self (familiar)-target/stranger (novel)-distractor condition than that of the stranger (novel)-target/self (familiar)-distractor pairs, while the distractor-rejection rate of the latter condition was faster than the former one. The result suggested that the familiar faces would promote the visual search when either identifying the target or rejecting the distractor. Further, as the number of distractors increases, the reaction time for the self-target/stranger-distractor condition eventually exceeded that for the stranger-target/self-distractors, supporting the advantage of familiar distractors in visual search task. Thus, the aforementioned studies suggested a benefit of familiar items as both targets and distractors in visual search task by using complex visual stimuli with high perceptual load.

Consequently, according to the perceptual load theory of attention, the contribution of the familiarity of target or distractor to the familiarity effect may depend on the perceptual load of the visual items. The perceptual load theory assumes that the perceptual processing is subject to attentional limited capacity and may not be controlled voluntarily at the early stage of perception. Thus, perceptual information might be automatically processed until the limited attentional resources is exhausted, and then the top-down cognitive control may reallocate more attentional resource to the relevant information^21–24^. As for the visual search task, searching for a letter target from homogeneous distractors may bear a low perceptual processing, with the target and distractor stimuli being identified in parallel (e.g.^10,13^). However, when the perceptual load of visual stimuli is high, such as a great many of different stimuli or complex perceptual discriminations (e.g.^17,19,20^), the attentional resource would be insufficient to process task-irrelevant stimuli in parallel. Thus the top-down cognitive control may allocate more attentional resource to the visual target, leading the visual search efficiency to be facilitated by the familiar target. Hence, the impact of target and distractor on the familiarity effect in visual search might be modulated by the perceptual load.

## The present study

To summarize, although Malinowski and Hübner^13^ proposed that visual search efficiency would be only related to the familiarity of distractors, these results have failed to be repeated by other studies with diverse visual materials (e.g.^9,16,25^). According to previous studies, the controversy on the mechanism of familiarity effect implies two primary theoretical issues to be addressed. For one thing, the critical criteria of perceptual load of visual materials need to be specified to disclose whether the perceptual load would modulate the impact of target and distractor on the familiarity effect in visual search. For the other thing, with the advantage of familiar distractors in visual search, what are the factors that would influence whether searching for a target among familiar distractors is an efficient process? Consequently, the present study attempted to explore the above issues by manipulating the visual perceptual load when searching the familiar or novel target among familiar distractors.

The present study used Chinese characters as the searching items to investigate the efficiency of searching target among familiar distractors. Chinese characters as a kind of linguistic symbol could be a special type of searching materials to examine the familiarity effect^25–27^. On the one hand, unlike the letters in alphabetic language, individual Chinese characters usually bear practical semantic meaning. As the letters may be distinguished from each other primarily by visual perceptual features or structures, the semantic meaning of Chinese characters involved in the visual search may influence the search efficiency by increasing the perceptual load. However, the vertical-structured “古” and the horizontal-structured “叶” used in research of Shen and Reingold^25^ might not be an appropriate pair. The search efficiency might benefit from the different structures between target and distractor^27^. Even if there was an efficient search in Shen and Reingold^25^, the conclusion cannot be drawn that the easy-grouped familiar distractors gave rise to the familiar effect. On the other hand, unlike the complex images that may elicit semantic processing during the visual search, the activation of the semantic meaning of over-learned Chinese characters could be a more automatic and rapid process^26,28^. The spontaneous semantic processing of Chinese characters may lead a lower perceptual load, compared to the complex visual materials.

**Figure 2:**
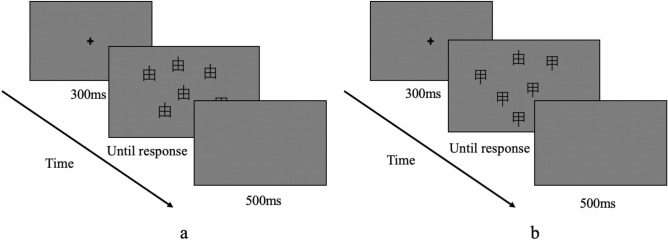
Timeline showing the process for a single visual search trial in Experiment 1. (**a**) Searching for “甲” from “由”. (**b**) Searching for “由” from “甲”.

Thus, the Chinese characters “甲” and “由” were served as the searching items for native Chinese speakers in Experiment 1 to first explored whether searching a familiar target from familiar distractors is an efficient search as seen in Malinowski and Hübner^13^ (see Fig. 2). “甲” and “由” are bilaterally symmetrical and commonly used in modern Chinese, while they afford completely different semantic meanings and pronunciations^29^. Their difference in visual perception only lies in the directions of extension of the vertical stroke in the middle of the Chinese character “田”. The Chinese character “由” can be regarded as a mirrored character of “甲” flipped around the horizontal axis. In addition, the characters “甲” and “由” matched the letters N and И from the perspective of visual perception. Therefore, our hypothesis for Experiment 1 is that searching for “由” from “甲” or searching for “甲” from “由” are inefficient when the target is absent, while it may turn to be efficient when the target is present.

## Experiment 1

### Results

When error rates of visual searching of all participants were lower than 10%, the search was deemed effective. Trials in which RT was out with three standard deviations under each condition are excluded. Only those RTs of accurate responses were submitted into the analyses. Averaged RTs were examined by repeated-measures analyses of variance (ANOVA). Greenhouse–Geisser corrected *p* values were reported for effects with two or more degrees of freedom in the numerator. Descriptive statistical results pertaining to Experiment 1 were summarized in Table 1.

**Table 1:** RTs and error rates in Experiment 1.

Target	Target presence	Set size	RT (ms)	SD	Error rates (%)
甲	Present	6	733.01	206.15	2.39
		8	804.32	233.76	3.51
		10	864.01	264.65	3.51
	Absent	6	950.07	310.13	0.19
		8	1113.73	381.32	0.74
		10	1187.85	440.04	0.73
由	Present	6	835.82	295.35	4.62
		8	926.25	341.86	3.31
		10	987.33	427.08	6.27
	Absent	6	1098.41	468.60	1.66
		8	804.32	563.80	1.11
		10	1436.64	624.53	1.29

#### RT

A target type × set-size ANOVA on the mean target-present RTs revealed all main effects to be significant and interaction effect to be non-significant: task type, F (1, 10) = 24.96, p < 0.001, ηp^2^ = 0.71; set size, F (2, 20) = 39.13, p < 0.001, ηp^2^ = 0.80. For target-present trials, the RT of searching for “甲” (M = 798.08 ms, SD = 162.66) was significantly shorter than that of searching for “由” (M = 911.71 ms, SD = 198.34). The ANOVA of the target-absent RTs indicated the same pattern of effects: task type, F (1, 10) = 35.25, p < 0.001, ηp^2^ = 0.78; set size, F (2,20) = 61.96, p < 0.001, ηp^2^ = 0.86. The interaction was not significant. For target-absent trials, the RT of searching for “甲” (M = 1084.42 ms, SD = 271.05) was also significantly shorter than that of searching for “由” (M = 1261.28 ms, SD = 331.80). The effect of set size and the non-significant interaction reflected the fact that the search slopes were overall non-flat and not differed between the target type conditions. A further exploration was conducted to analyze the search slopes directly.

#### Visual search efficiency

**Figure 3:**
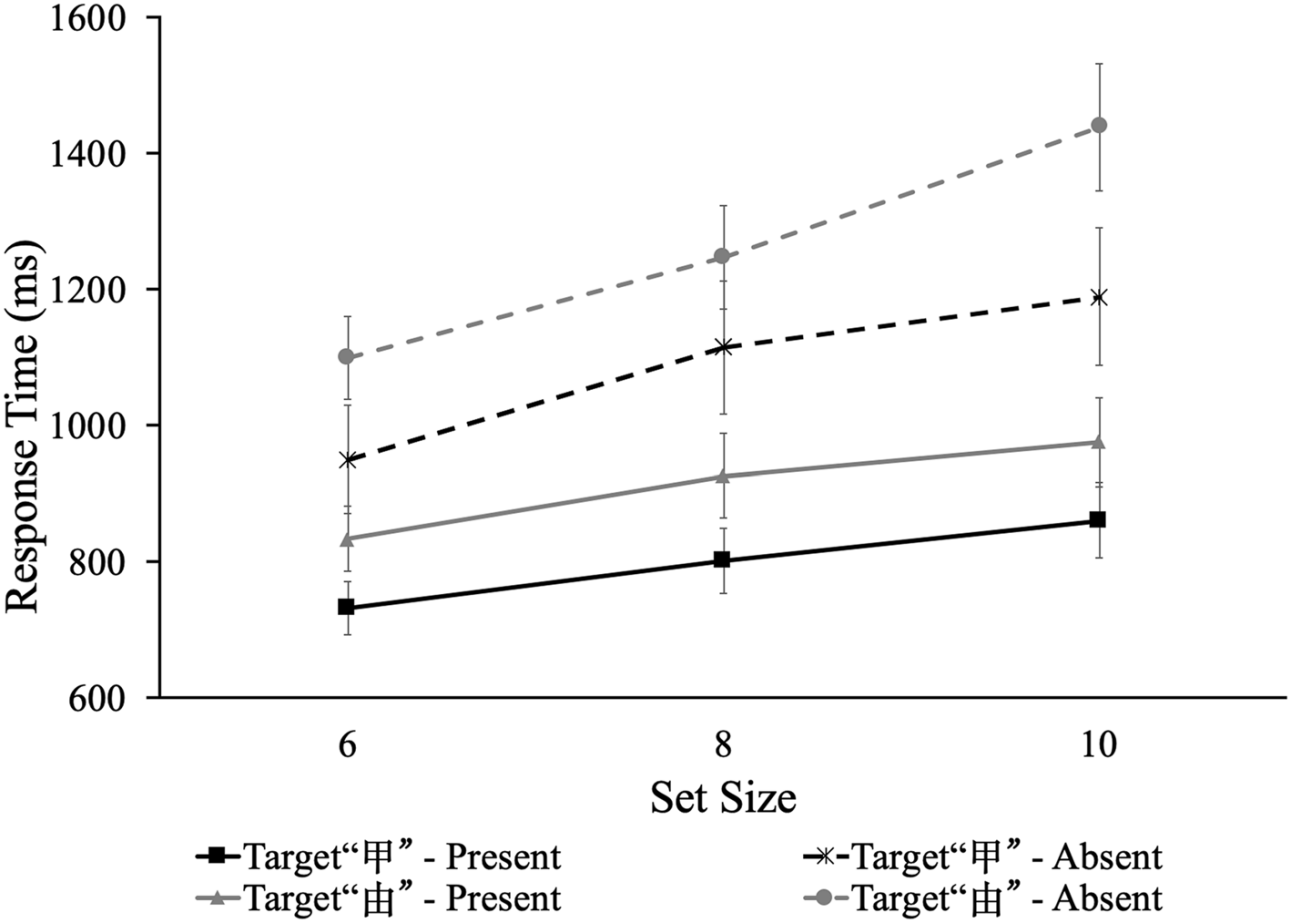
Mean RTs as a function of set size in Experiment 1. Error bars denote the standard error.

For each target, search slope of each participant for target-present and target-absent trials were produced separately by a linear regression of RT on set size as *RT* = *b*_*1*_ + *b*_*2*_ × *set size*. The trends in RT with set size are shown in Fig. 3. The search slopes differed from zero under all conditions, all *t*s > 5.00, all *p*s < 0.001, as shown in Table 2. Furthermore, a 2(target type) × 2(target presence) repeated measure ANOVA was performed. The results demonstrated significant main effect of target presence, *F* (1,10) = 31.61, *p* < 0.001, η_*ρ*_^2^ = 0.76. The slope of the visual search function for the target present condition (*M* = 33.94 ms/item, *SD* = 18.87) was significantly smaller than that for the target absent condition (*M* = 72.44 ms/item, *SD* = 32.94). Slopes of the visual search function showed significant difference between searching for “甲” and for “由”, *F* (1,10) = 10.24, *p* < 0.01, η_*ρ*_^2^ = 0.51. The slope of searching for “甲” (*M* = 45.98 ms/item, *SD* = 31.08) was significantly smaller than that of searching for “由” (*M* = 60.40 ms/item, *SD* = 33.79).

**Table 2:** Mean search slopes (in ms/Item) and intercepts (in ms) with standard errors (in brackets), for target-present and target-absent trials in Experiment 1.

Target	Present	Absent
甲	32.20*** (17.58)	59.74*** (36.08)
由	35.68*** (20.78)	85.12*** (24.94)

Asterisks indicate that the respective slope differs significantly from zero. *p < .05, **p < .01, ***p < .001.

### Discussion

The present results did not repeat the finding in Malinowski and Hübner^13^ by showing that searching for a familiar target from familiar distractors with the Chinese characters is an inefficient search. Further, visual search efficiency for the present target condition is higher than that under the absent target condition. These results support our hypothesis that the searching is more efficient when the target is present than that when the target is absent. However, the present study showed that the search efficiency for task of searching “甲” from “由” is higher than that for searching “由” from “甲”. Although both were commonly used Chinese characters, participants may have different familiarities for the character “甲” and “由”.

## Experiment 2

**Figure 4:**
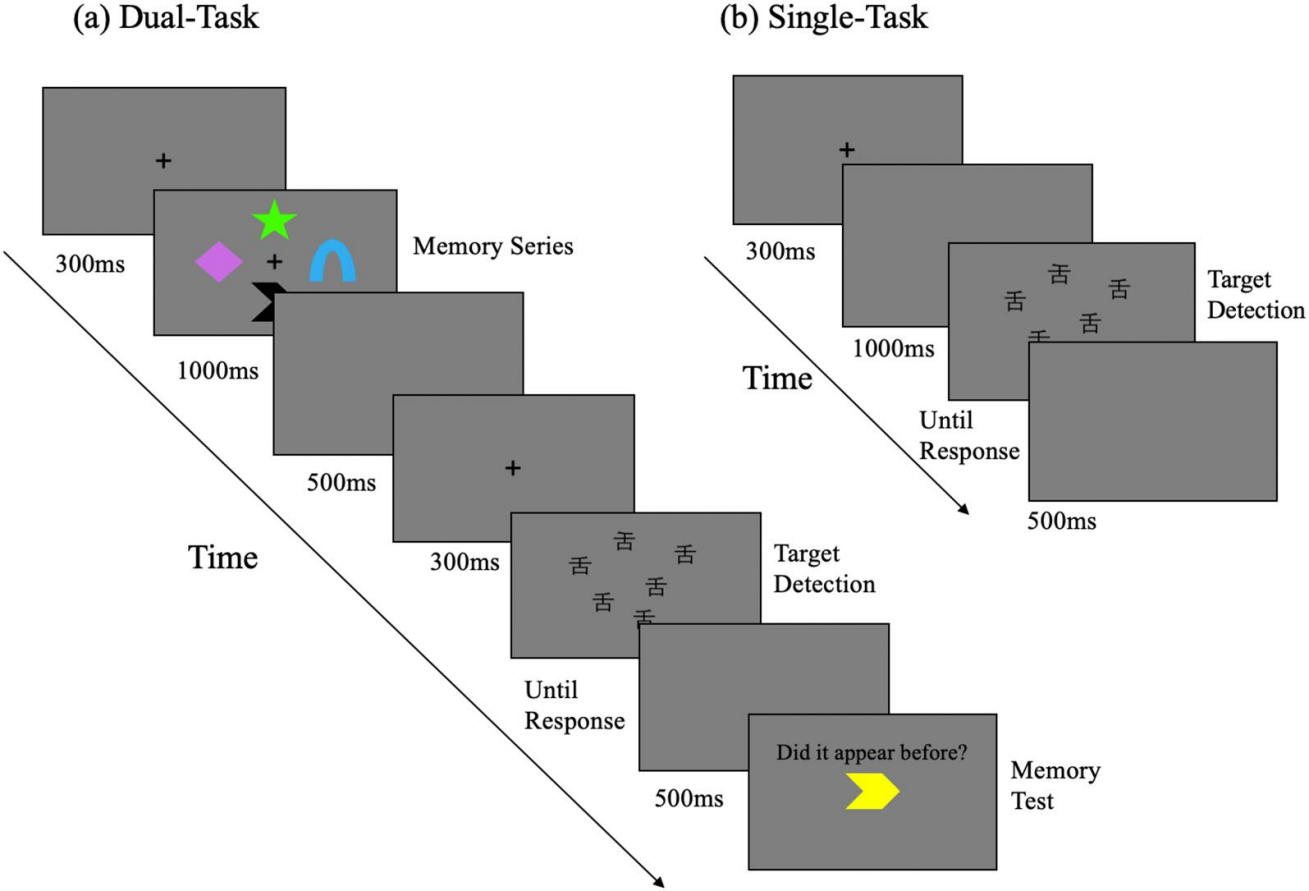
Timeline showing the procedure for a single trial in Experiment 2. (**a**) The dual-task condition including a visual search task and a memory task. (**b**) The single-task condition is a purely visual search task.

Experiment 1 examined the search efficiency with specific Chinese characters when the target and distractors were both familiar to subjects. The results demonstrated that seeking familiar target from familiar distractors was an inefficient search when involving probably automatic semantic processing. To further explore the impact of familiarity of target on familiarity effect, Experiment 2 focused on the visual search for a novel target from homogeneous familiar distractors, as well as the effect of increasing perceptual and working memory load. Experiment 2 used Chinese character “舌” and its mirror image, which is not a real Chinese Character, as the searching items (see Fig. 4). “舌” is also a frequently used character in modern Chinese^25^. Hence, a cross-study comparison between the familiar-target/familiar distractor and novel-target/familiar distractor condition would be performed by comparing the data in Experiment 1 and Experiment 2. According to the theoretical account that only the familiarity of distractor matters for familiarity effect, the efficiency of searching familiar or novel target among familiar distractors would not differ from each other.

As addressed in the introduction section, the controversy on visual search efficiency for various target-distractor combinations might be resulted from the different levels of perceptual load. Recent work highlights the intimate connection between visual working memory (vWM) and the perceptual load^30–32^. For example, increasing visual working memory load would reduce the processing of task-irrelevant information^32^. The modeling result of Kyllingsbæk et al.^31^ suggested that the perceptual load effect could only be explained by incorporating the processing and storage capacity constraints of vWM. Comparable with the perceptual load effect, evidence from dual-task research suggested that loading visual working memory maintenance increased the demand on sensory representation and diminished the perception of low-priority stimuli^30^. Therefore, we attempted to manipulate the perceptual load of searching task by conducting a dual task paradigm in Experiment 2.

Further, it may not be appropriately to change the perceptual load by using other Chinese characters, as the processing of over-learned characters for native speakers could be quite similar. The dual task paradigm with vWM task provided an opportunity to increase the perceptual load from an external source and keep it through the subsequent visual search. As Fig. 4 illustrates, participants would memorize colors and shapes to increase the perceptual and vWM load before executing visual search task. The perceptual and conceptual processing of colors and shapes may interfere with the processing of searching items, showing an impact on the search efficiency if this is the case. Otherwise, if the performance of dual task condition does not differentiate from that of single task condition, then external perceptual load may have little influence on the search efficiency.

In addition, the impact of vWM load can also be reflected in the search slopes of target-present trials and target-absent trials. The efficiency of searching novel target would be decreased by the extra vWM load, while the efficiency of rejecting distractors might not be affected, as the processing of overlearned Chinese characters may be automatic and effortless^32,33^. In this case, we hypothesized that visual search efficiency in dual-task condition would be lower than that in single-task condition when the target is present, but be similar when the target is absent.

### Results

When error rates of visual searching of all participants were lower than 10%, the search was deemed effective. Trials in which RT was out with three standard deviations under each condition were excluded. There were 23 valid datasets remaining. Only those RTs of accurate responses were submitted into the analyses. Descriptive statistical results pertaining to Experiment 2 are summarized in Table 3.

**Table 3:** Response time and error rates in Experiment 2.

Task type	Target presence	Set size	RT (ms)	SD	Error rates of visual search task (%)	Error rates of memory task (%)
Single	Present	6	1103.10	256.83	4.67	–
		8	1171.96	304.91	4.43	–
		10	1282.52	326.31	5.09	–
	Absent	6	1486.46	327.10	1.31	–
		8	1728.47	429.31	1.76	–
		10	1931.96	491.51	2.45	–
Dual	Present	6	1524.97	570.41	5.76	26.92
		8	1597.76	590.99	3.27	26.98
		10	1705.62	665.84	5.22	30.42
	Absent	6	1943.83	580.50	1.23	27.08
		8	2117.93	665.52	1.24	26.77
		10	2328.91	690.92	2.19	30.21

#### RT of visual search task

For target-present trials, a task type (2) × set-size (3) ANOVA on mean RTs showed the significant main effects of task type, *F* (1,22) = 15.56, *p* = 0.001, *η*_*ρ*_^2^ = 0.41 and set size, *F* (2,44) = 17.94, *p* < 0.001, *η*_*ρ*_^2^ = 0.45. For target-present trials, the RT of single-task condition (*M* = 1164.16 ms, *SD* = 305.31) was significantly shorter than that of dual-task condition (*M* = 1596.74 ms, *SD* = 616.66). Interaction was not significant. The results of target-absent trials also revealed the same pattern of effect for task type, *F* (1,22) = 10.76, *p* = 0.003, *η*_*ρ*_^2^ = 0.33 and set size, *F* (2,44) = 93.36, *p* < 0.001, *η*_*ρ*_^2^ = 0.81. For target-absent trials, the RT of single-task condition (*M* = 1720.00 ms, *SD* = 449.98) was significantly shorter than that of dual-task condition (*M* = 2138.07 ms, *SD* = 665.53). Interaction was not significant. The effect of set size and the non-significant interaction between task type and set-size reflect the fact that the search slopes (1) were overall non-flat and (2) did not differed between the single-task and dual-task condition. A further exploration was conducted to analyze the search slopes directly.

#### Visual search efficiency

For each task type, search slope of each participant for target-present and target-absent trials were calculated separately by a linear regression of RT on set size. One-sample directed *t-*test against zero showed the search slopes differed from zero under all conditions, all *t*s > 4.14, all *p*s < 0.001, as shown in Table 4. For target present trials, independent-sample *t*-test was performed between the slopes of searching familiar target among familiar distractors (Experiment 1) and those of searching novel target among familiar distractors (single-task condition in Experiment 2). The results indicated that the search efficiencies were not different between searching for “甲” among “由” (or searching for “由”among “甲”) and searching for mirror “舌” among “舌”.

**Table 4:** Mean search slopes (in ms/item) and intercepts (in ms) with standard errors (in brackets), for target-present and target-absent trials in Experiment 2.

Target	Present	Absent
Single	38.60*** (6.79)	112.42*** (36.08)
Dual	42.73*** (10.31)	96.11*** (24.94)

Asterisks indicate that the respective slope differs significantly from zero. *p < .05, **p < .01, ***p < .001.

**Figure 5:**
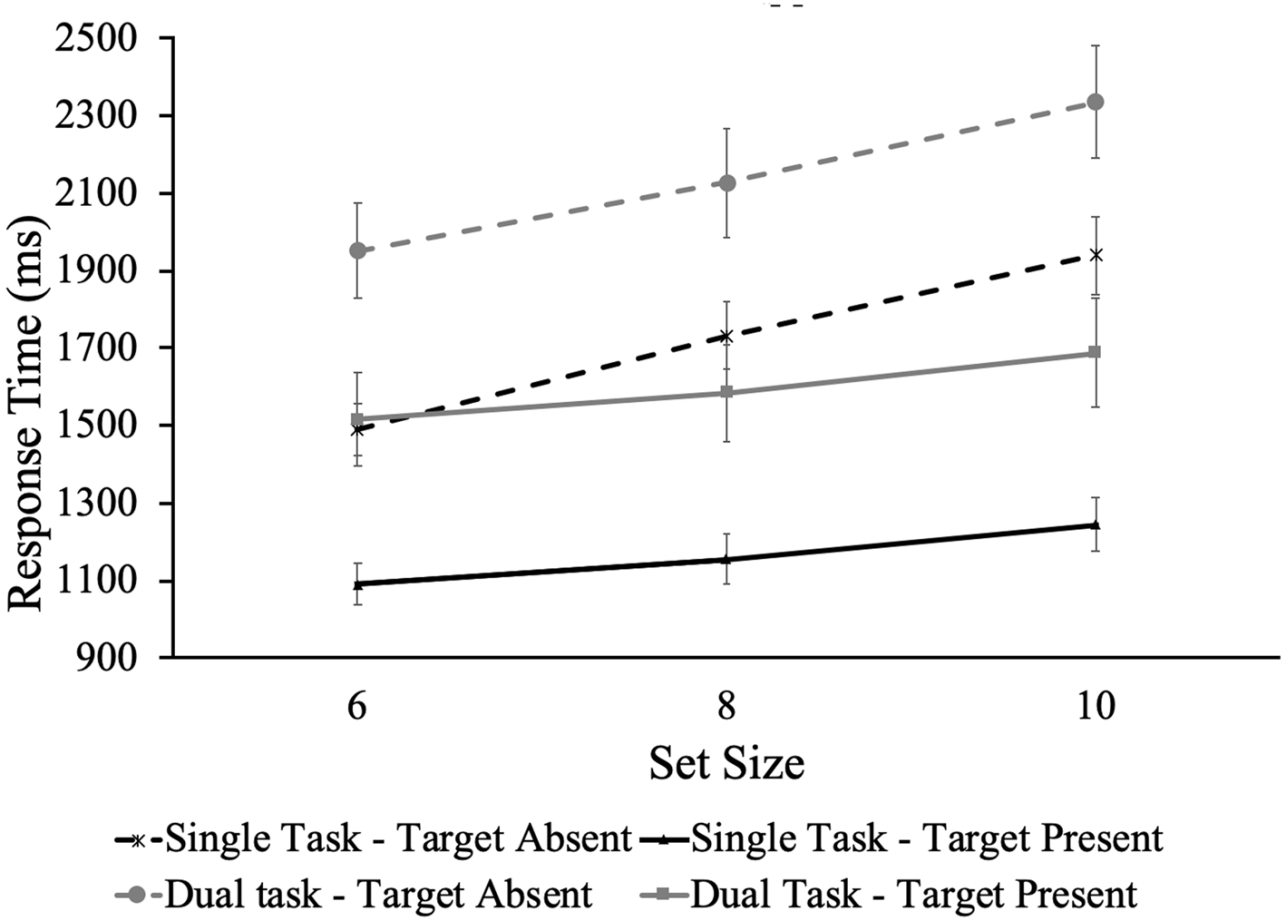
RTs as a function of target presence and set size for the different tasks in Experiment 2. Error bars denote the standard error.

Furthermore, a 2(task type) × 2(target presence) repeated measure ANOVA was performed. The results only demonstrated a significant main effect of the target presence, *F* (1,22) = 35.56, *p* < 0.001, *η*_ρ_^2^ = 0.62. The slope when the target was present (*M* = 40.67 ms/item, *SD* = 41.44) is significantly smaller than that when the target was absent (*M* = 104.26 ms/item, *SD* = 55.42). The effect of task type is not significant and there is no significant interaction between task type and target presence (see Fig. 5).

#### Error rate

The average error rate of visual search tasks was 3.19%, showing no trade-off between accuracy rate and response time. Repeated-measured ANOVA under conditions of 2 (task type) × 2 (target presence) × 3 (set size) was conducted on the error rate of visual search tasks. The results showed a significant main effect of the target presence, *F* (1,22) = 24.26, *p* < 0.001, *η*_*ρ*_^2^ = 0.52. The error rate of target present condition (*M* = 4.70%, *SD* = 0.04) was significantly higher than that of target absent condition (*M* = 1.70%, *SD* = 0.03).

The average error rate of memory tasks was 26.09%. By performing repeated-measured ANOVA under conditions of 2 (target type) × 2 (target presence) × 3 (set size) on memory tasks of the subjects, none of main effects and interactions were significant.

### Discussion

The results of Experiment 2 suggested that searching for mirror “舌” is inefficient and did not benefit from the novel target. The search was easier when the target present than the target is absent under both single- and dual-task conditions. The effect of vMW load was not supported, as no interaction was found in search efficiency between task type and the target presence. The extra vWM load may not influence the visual search efficiency when searching for the novel target from familiar distractors.

## General discussion

The present study investigated the impact of target on familiarity effect with Chinese characters by manipulating the perceptual load. Experiment 1 suggested that it is inefficient to search for a familiar target among familiar distractors. This finding is contrary to the research using letter N and И^13^ or characters “古” and “叶”^25^, but consistent with those using materials with complex perceptual features^9,16^. Further, Experiment 2 found no effect of extra vWM load on the efficiency of search a novel target from familiar distractors. Combining the results in Experiment 1 and 2, the search efficiency did not show difference between searching for familiar and for novel target when the distractors were familiar based on the perceptual load of processing Chinese characters. This was in line with the theory that the familiarity of target would not influence the familiarity effect^13^.

Previous studies used letters as experimental materials showed an efficient search when searching a familiar target from familiar distractors, while the search has been inefficient when we use Chinese characters which share similar visual perceptual features but afford different semantic meanings. The orthographic similarity between the target and the distractor may decrease the efficiency of the search^2,34^. Although the visual feature similarity between the target and distractor yield the familiarity effect^16^, the efficiency of searching for familiar target among familiar distractors would also be influenced by the remarkable similar visual features of stimulus. In order to accomplish a very efficient visual search driven by the familiarity, the distinctness in visual features between the target and distractor should meet strict criteria to ensure the target popping out from distractors.

Further, the automatically semantic processing of Chinese characters may also decrease the efficiency of searching the target from familiar distractors, especially when interacted with the similar visual features. We used the mirror “舌” as the novel target to compare with the efficiency of searching familiar target from familiar distractors. The mirror image did not actually afford meaningful conceptual information and share little semantic similarity with the distractors. The results showed again no difference in search efficiency between the novel-familiar and familiar-familiar pairs. This finding suggests that the present perceptual features might not be salient enough to pop out when the distractors involve the automatic semantic processing during the visual search. In addition, the perceptual features of novel target similar to familiar distractors may also prime the semantic or conceptual representation of the distractor, leading to the comparable efficiency to that of searching for the familiar target. Shen and Reingold^25^ supposed that the automatic and holistic processing for familiar characters might inhibit people from capitalizing on certain cues or features, such as the orientation of a specific line, thereby reducing the search efficiency.

Based on perceptual load theory, the present study offers a new perspective to understand the divergence of previous studies about how the familiarity of target or distractor impact the visual search efficiency. Specifically, studies used simple stimuli and homogeneous distractors did not show the influence of target familiarity, while those employed materials with complicated features benefit from familiar targets. Previous research also suggested that the vWM load of irrelevant information under dual-task conditions would not affect attention allocation in a visual search task^35,36^. Therefore, it is possible that the promotion of a novel target on attention attraction remains stable with extra vWM memory load. In addition, the processing of the mirror image of “舌” might require relatively low perceptual load, leading little impairment of the increased visual working memory load on familiarity effects. The present study conducted an extra vWM task to intimate the perceptual load level of performing a difficult visual search task. Without showing any evidence of the effect of the extra load, it may imply that the perceptual load coming from visual search items themselves would impact more on the search efficiency.

One further issue about the effect of the extra working memory task is that the theory model of working memory is proposed to involve both visual working memory for storing perceptual features and spatial working memory for storing spatial information about objects or environments^37–39^. During visual search, attention needs to shift from one position to another in order to capture the target, while the previously attended location will be inhibited to gain attention again^36,40^. There is the rehearsal mechanism to memorize the locations of already attended items, which can be hindered by loading spatial working memory^36,41^. Thus one extra spatial memory task would usually impair the visual search. However, the present study stressed whether the change of perceptual load would modulate the visual search efficiency. We did not intend to interfere in the visual search process by impeding the attention shift process. Instead, we attempted to conduct the state of high perceptual load by loading the visual working memory without altering the visual search task itself. In this way, we may address the divergence between studies that using different materials in visual search from the perspective of the perceptual load. Further studies can be conducted from two perspectives to explore the effect of working memory on familiarity effect in visual search task. One route is to adopt short phrases as visual search materials to make the semantic processing to be comparable to the object images used in Mruczek and Sheinberg^17^. The other one is to avoid verbal encoding during the memory task, such as using irregular patterns or location memory task instead, to examine the effect of visual working memory on familiarity effects.

To conclude, the present study lends supports to the theoretical account that the familiarity effect in visual search would not be influenced by the target familiarity involving either high or load perceptual load. Further, when we used the Chinese characters which involve automatically semantic process, the visual search is not as efficient as the materials with salient visual distinctions. That understanding how familiarity shape visual search may reveal how our daily experience formed mental representations for outside world and optimize our performance. It is of theoretical and practical significance to study visual search asymmetry based on different familiarities to targets and distractors. In theory, visual search asymmetry emphasizes the relationship between distractors and targets in a visual search. The research on the topic can provide the understanding of the allocation of attention and cognitive resources between the target and distractors in visual search. It is also helpful for understanding the difference in mechanisms used for generating a parallel search and a serial search. In practice, how to improve the efficiency of a visual search is vital in the design of interactive interfaces and the study of visual search asymmetry provides design guides for interface designers. For example, while considering how to enable users to locate important information faster, designers should also pay attention to whether the features of disturbance information would affect the search for the target information.

## Methods

### Experiment 1

#### Participants

Eleven (9 females, M = 19.64-year-old, SD = 1.43) right-handed undergraduates with Chinese as their native language voluntarily participated in the experiment. All participants had normal or corrected visual acuity. Participants signed the informed consents before the experiment and were paid in monetary form. This study was approved by the Institution Review Board of Department of Psychology, Sun Yat-Sen University and was performed in accordance with relevant ethical guidelines and regulations.

#### Materials

Stimuli were shown on the HP ProDisplay P231 LED display screen. The screen measured 23 inches (584 mm), with a resolution of 1920 × 1080 pixels, and refresh frequency of 60 Hz. The Song typeface characters “甲” and “由” at a 32-point font size were randomly distributed in a square grey background with subtended angular dimensions of 13° × 13°. The participants were seated about 560 mm away from the screen.

#### Design

The experiment used a target type (“甲” or “由”) × target presence (absent or present) × set size (6, 8, or 10 items) within-subject design. The target type variable separately corresponded to the task of searching for “甲” from “由” (target “甲”) and searching for “由” from “甲” (target “由”). The target presence variable indicated whether the target appeared or not. The target appeared randomly in half of the total trials. The set size variable was the number of stimulus items, with levels thereof being 6, 8, or 10 distributed randomly in the grey background.

There were 600 trials in total with 50 trials for each of the twelve experimental conditions. The total trials were divided into two blocks for each target type (searching for “甲” from “由” or searching for “由” from “甲”) and the order of the blocks was counterbalanced across participants. Participants completed ten practice trials before each block to get familiar with the task. The dependent variables were the error rate and response time (RT) of the correct trials.

#### Procedure

The MATLAB 2015b and Psychtoolbox-3 were used to program the experimental procedures. Participants were randomly assigned to the group that searching for “甲” from “由” first (see Fig. 2a) or searching for “由” from “甲” first (see Fig. 2b). The experimental procedure is shown in Fig. 2. Before the visual search series appeared, a fixation was presented at the center of the screen for 300 ms. Then as soon as the stimuli appeared, participants were required to seek the target from multiple distractors by pressing specific keys quickly and accurately. After participants responded, the procedure turned to a blank screen for 500 ms, with an interval of 1000 ms between trials.

### Experiment 2

#### Participants

Twenty-four undergraduate students (16 females, *M* = 20.4-year-old, *SD* = 2.12) volunteered for participation in the experiment, excluding one male participant whose error rate in visual search task was greater than 10%. All participants were native Chinese speakers and had normal or corrected visual acuity. Participants signed the informed consents before the experiment and were paid in monetary form.

#### Materials

The setup of devicein Experiment 2 matched Experiment 1. The screen background was grey and the graphs for memory tasks were the eight graphs which were used in working memory studies (see examples in Fig. 4, e.g.^42^). Each graph had seven saturated colors, including red (RGB: 255, 0, 0), blue (RGB: 0, 0, 255), purple (RGB: 138, 43, 226), green (RGB: 0, 255, 0), yellow (RGB: 255, 255, 0), black (RGB: 0, 0, 0), and white (RGB: 255, 255, 255). Each graph measured 2.35° × 2.35° and the central point was 3.44° from the centre of the screen. Song typeface character “舌” and its mirror image at a 32-point font size were used as materials in visual search tasks, which were randomly distributed within a square area measuring 13° × 13°.

#### Design

The experiment used a task type (single or dual-task) × target presence (absent or present) × set size (6, 8, or 10 items) within-subject design. For the dual-task condition, participants carried out a memory task and a visual search task, while participants only performed the visual search task in single-task condition. The manipulation of target presence and set size variable matched Experiment 1.

There were 600 trials in total with 50 trials for each of the twelve experimental conditions. The total trials were divided into two blocks for each task type (single- or dual-task) and the order of the blocks was counterbalanced across participants. Participants completed ten practice trials before each block to get familiar with the task. The dependent variables were the error rate and response time (RT) of the correct trials.

#### Procedure

The experimental procedure is illustrated in Fig. 4. For the dual-task group, after presenting the fixation for 300 ms, the memory series including four graphs were presented for 1000 ms. Participants were asked to memorize colors and shapes of the four graphs with different colors and shapes. Previous research has demonstrated that memorizing four targets can saturate working memory (^43,44^). Then, the visual search series was shown after showing a blank screen for 500 ms and the fixation for 300 ms. After participants responded, the screen turned blank for 500 ms. Participants were required to search for the target, namely a mirrored “舌” among multiple distractors. A blank screen was presented for 500 ms after completion of the visual search tasks and then a memory test was conducted. Participants would judge whether the presented graph had appeared before. There was a 50% chance that the presented graph had been shown in the learning phase. Once participants made their responses, the single trial finished. For the single-task condition, participants only performed the visual search task, in which 1000 ms under dual-task conditions for presenting memory serial stimuli was replaced with a blank screen for 1000 ms. After presenting the fixation for 300 ms, the visual search task was conducted. After each response, the screen went blank for 500 ms and the interval between trials was 1000 ms for both dual- and single-task group. One issue needs to note is that the time for the dual and single task were not equivalent, as the dual-task of vWM process was used to increase the cognitive load. The extra working memory task would naturally prolong the dual-task, although we have tried to equate the time for dual and single task by using an 1000 ms interval before presenting the searching screen.

## Data Availability

The datasets generated during and/or analyzed during the current study are available from the corresponding author on reasonable request.
